# Dual bio-active factors with adhesion function modified electrospun fibrous scaffold for skin wound and infections therapeutics

**DOI:** 10.1038/s41598-020-80269-2

**Published:** 2021-01-11

**Authors:** Jianhang Jiao, Chuangang Peng, Chen Li, Zhiping Qi, Jing Zhan, Su Pan

**Affiliations:** 1 Department of Orthopedic Surgery, The Second Hospital of Jilin University, Ziqiang Street No. 218, Changchun, 130041 Jilin People’s Republic of China; 2 Department of Gastroenterology, First Hospital of Jilin University, Jilin University, 71 Xinmin Street, Changchun, 130021 Jilin People’s Republic of China

**Keywords:** Biomaterials - proteins, Biomedical materials, Tissues

## Abstract

Electrospun fibrous scaffolds combined with bioactive factors can display impressive performance as an ideal wound dressing, since they can mimic the composition and physicochemical properties of the extracellular matrix (ECM). The aim of this study was to fabricate a new composite biomaterial (IGF1-DA and Os-DA-modified PLGA electrospun fibrous scaffold) for wound healing, using a rat model for experimental evaluation. A small pentapeptide tag composed of DA–Lys–DA–Lys–DA residues was introduced into insulin-like growth factor 1 (IGF1) and the antimicrobial peptide Os to prepare IGF1 and Os modified with 3,4-dihydroxyphenylalanine (DA) (IGF1-DA and Os-DA). The designed chimeric growth factor and antimicrobial peptide could successfully anchor to PLGA electrospun fibrous scaffolds, and the growth factor and antimicrobial peptide could be controllably released from the electrospun fibrous scaffolds. The results showed that the IGF1-DA and Os-DA-modified PLGA electrospun fibrous scaffolds (PLGA/Os-DA/IGF1-DA) exhibited high hydrophilicity and antimicrobial activity; moreover, the porous network of the scaffolds was similar to that of the natural ECM, which can provide a favourable environment for BALB/C 3T3 cells growth. The in vivo application of PLGA/Os-DA/IGF1-DA electrospun fibrous scaffolds in rat skin wounds resulted in improved wound recovery and tissue regeneration rate. The experimental results indicated that the IGF1-DA and Os-DA could effectively bind to PLGA electrospun fibrous scaffolds, promote wound healing and prevent infection in rats, thereby suggesting that PLGA/Os-DA/IGF1-DA electrospun fibrous scaffolds have a wide application value in the field of skin wound repair.

## Introduction

The main purpose of skin tissue engineering is to prepare wound dressings with good biological activity and tissue repair capabilities^1,2^. In recent years, nanofiber scaffolds made of various natural and synthetic compounds have been widely used in the research of wound repairs^3,4^. Electrospun scaffolds have received much attention, primarily because of the simple electrospinning process, which does not require expensive or complex fabrication instruments, and the scaffolds porous structure similar to the human native extracellular matrix (ECM)^5^. The highly porous three-dimensional (3D) structure of nanofibers can facilitate the exchange of cellular nutrients and create an ideal cellular microenvironment for wound healing. Furthermore, electrospun fibrous scaffolds also have several advantages, including an extremely high specific surface area, adjustable porosity, controllable size and shape and the ability to regulate the nanofiber functions by controlling the nanofiber composition^6^. More importantly, during epidermal reconstruction, the ECM structure of the electrospun fibrous scaffold plays a crucial role in the coordination of various biological changes, such as changes in cells, ECM components and signals formation. The above advantages, which greatly improves the skin wound treatment efficiency, make electrospinning a widely used method for wound dressing production. Furthermore, to functionalise the electrospun fibrous scaffold for the promotion of skin wound healing, it can be loaded with several bioactive factors, such as proteins, peptides and small-molecule drugs^7–9^. Therefore, electrospun fibrous scaffolds have remarkable advantages in both ECM-biomimetic structures and the ability to adsorb bioactive factors and have thus emerged as essential applications in the field of skin wound repair.

Skin wound healing is a complex process that can be affected by multiple contributing factors, such as ischaemia and infection^10^. Previous studies have found that the main cause of ischaemia is the decreased level of growth factors in the traumatic environment^11^. The initiation of wound repair is associated with the release of several growth factors, such as the platelet-derived growth factor, basic-fibroblast growth factor and transforming growth factor^12–14^. Moreover, bacterial infections can also severely affect wound healing, as the exposed wound tissue provides an appropriate environment for rapid bacterial growth and proliferation. Once bacteria begin to grow on the wound surface, they will multiply and form colonies and invade deeper tissues or blood circulation through the wound, which can result in multiple complications, including septicaemia, organ necrosis and even death^15^. Thus, many researchers have introduced a growth factor or an antibacterial factor into electrospun fibrous scaffolds, to improve the skin wound repair efficiency^8,16,17^. Among all growth factors, insulin-like growth factor 1 (IGF1) is a widely used tissue repair growth factor and is widely considered to have a strong ability to promote the proliferation of many cell types, such as those of the skeletal muscle, cartilage, bone, liver, kidney, neural tissues, skin, haematopoietic tissues and lung^18,19^. Moreover, locally delivered IGF1 has been found to promote wound healing in senescent mice and in a rat tissue ischaemia model^20^. Furthermore, during the wound healing, IGF1 stimulates collagen synthesis in fibroblasts, stimulates the proliferation of fibroblasts and keratinocytes and may also be involved in angiogenesis^21^. Exogenous IGF1, applied alone or combined with biomaterials having other bioactive factors, has been demonstrated to have a positive effect on wound healing^20,22^. In terms of inhibiting bacterial infections, the most commonly used method is to introduce antibiotics to enhance the antibacterial properties of skin dressing. However, bacterial antibiotic resistance, due to the antibiotic overuse, severely limits antibiotic usage in the prevention of wound infections. Antimicrobial peptides (AMPs) are an important part of the innate immune system of organisms and can reasonably solve the problem of bacterial resistance^23^. The short C-terminal peptide Os, from OsDef2, a defensin in the midgut of the soft tick *Ornithodoros savignyi*, has been studied in the literature^24^. The main antibacterial effects of Os is the destruction of the bacterial cell structure and the indentation of the cell membrane. Moreover, Os has been shown to be bactericidal to both Gram-positive and Gram-negative bacteria^25^. It has also been found to have no effect on mammalian cell activity and possess antioxidant activity^24^. Given the above findings, the use of IGF1 and Os combination to improve the antibacterial properties and tissue repair ability of electrospun fibrous scaffold has attracted our attention.

Various delivery systems have been considered for incorporating growth factors or AMPs in polymer materials. Among such systems, the surface immobilisation method has attracted much attention because it avoids the destruction of bioactive factor activity. However, the poor surface hydrophilicity of the polymer and the lack of functional groups often result in lower bioactive factor loading efficiencies. In recent years, inspired by the strong adhesion ability of marine mussels to various materials, researchers have investigated the catechol ligand 3,4-dihydroxyphenylalanine (DA) as a coupling agent^26^. Recent studies have indicated that DA can be used as a coupling agent to bind bioactive factors on different materials^27^. Furthermore, in recent years, to accurately control functional molecules and avoid the complex process of the traditional DA surface immobilisation method, some studies have introduced site-specific DA into bioactive factors using the recombinant DNA technique^28,29^. The DA-modified bioactive factors were found to possess the adhesion ability of DA and could readily anchor onto various substrates surfaces. The immobilisation of the DA-modified bioactive factors on polymer materials surface could endow the polymer materials with stronger biological functions, which is an important step in the development of functional biomaterials. Thus, DA-modified bioactive factors provide new insights and methods for the application of DA surface modification. Given the above reasons, we speculate that DA-modified growth factors and AMP can effectively bind on the electrospun fibrous scaffold and promote skin wound healing.

In this study, a small pentapeptide tag composed of DA–Lys–DA–Lys–DA residues was introduced into native-IGF1 (NAT-IGF1) and native-Os (NAT-Os) to prepare IGF1-DA and Os-DA. Then, the IGF1-DA and Os-DA were used for the surface bio-functionalisation of poly lactic-co-glycolic acid (PLGA) electrospun fibrous scaffolds. The biological activity and antimicrobial effect of the new electrospun fibrous scaffolds were characterised and analysed. Furthermore, the cell adhesion and proliferation of the different electrospun fibrous scaffolds were evaluated in vitro. Finally, we investigated the wound healing ability of the PLGA/Os-DA/IGF1-DA electrospun fibrous scaffolds in a full-thickness wound model.

## Materials and methods

### Reagents

The polymer PLGA (75:25, Mw = 15,000) was purchased from Sigma (Shanghai, China). The polypeptides Os, fluorescein isothiocyanate (FITC)-conjugated Os (FITC-Os), Os–Tyr–Lys–Tyr–Lys–Tyr (Os-Tyr^3^) and FITC-conjugated Os-Tyr^3^ (FITC-Os-Tyr^3^) were synthesised by GL Biochem (Shanghai) Co., Ltd. The organic compound 1,1,1,3,3,3-hexafluoroisopropanol (HFIP) was purchased from Shanghai Aladdin Co., Ltd. Dulbecco’s modified eagle medium (DMEM, high glucose) and fetal bovine serum (FBS) were purchased from Gemini (USA). Cell Counting Kit-8 (CCK-8) was purchased from Beyotime Co., Ltd. (“Supplementary Information”).

### Expression and purification of NAT-IGF1 and IGF1-Tyr^3^

Plasmids of NAT-IGF1-PET15b and IGF1-Try^3^-PET15b) were constructed by Sangon Co., Ltd (Shanghai, China). First, strains containing plasmids were cultured overnight, and then an extended culture was conducted at a scale of 1:100 at 37 °C (150 rpm). When the optical density OD_600_ reached 1.0, the cultures were induced to express the target protein by 1 mM IPTG, and the culture conditions were 22 °C, 180 rpm, and 12 h. The cultures were then centrifuged at 8000 rpm, 4 °C for 30 min to collect the bacteria. After the bacteria were resuspended by a lysis buffer, they were broken with an ultrasonic crusher for 10 min in an ice bath. The broken bacteria were centrifuged at 13,000 rpm, 4 °C for 30 min to collect the supernatant. The supernatant was purified by affinity chromatography (Ni-NTA). The purified NAT-IGF1 and IGF1-Try^3^ were desalted using a G25 desalination column. Afterwards, the NAT-IGF1 and IGF1-Try^3^ were analysed via sodium dodecyl sulphate–polyacrylamide gel electrophoresis (SDS-PAGE) and Western blotting (WB).

### Tyrosine hydroxylation

Tyr–Lys–Tyr–Lys–Tyr tag in IGF1-Try^3^, Os-Try^3^ and FITC-Os-Try^3^ can be transformed into the DA–Lys–DA–Lys–DA tag by the catalytic reaction of tyrosine hydroxylase. First, IGF1-Try^3^, Os-Try^3^ and FITC-Os-Try^3^ were prepared into 1 mg/mL using PBS. Then, IGF1-Try^3^, Os-Try^3^ or FITC-Os-Try^3^ (250 μg/ml), tyrosine hydroxylase (125 μg/ml) and ascorbic acid (62.5 mM) were mixed and reacted for 5 h at 25 °C. The PH of the reaction buffer was adjusted to 8.5 with a solution of 50 mM Tris–HCL (PH 8.5). After the above chemical reaction process, the tyrosine constituents in IGF1-Try^3^, Os-Try^3^ and FITC-Os-Tyr^3^ were converted to DA molecules.

### Preparation of electrospun fibrous scaffolds modified with IGF1-DA and Os-DA

First, 1.5 g PLGA was added to 10 mL HFIP, and the mixture was incubated at 60 °C for 6 h to accelerate the PLGA dissolution, and a 15% (w/v) PLGA solution was obtained for the preparation of electrospun fibrous scaffolds. Then, 2.5 mL syringes and 30G needles were used in the electrospun fibrous scaffold preparation. The distance from the needle to the receiving plate was set as 15 cm, and the voltage was set as 16 kV. The extrusion speed of the PLGA solution was set as 0.15 mL/min. The prepared electrospun fibrous scaffolds were dried in a vacuum drying box for 48 h to remove the solvent. Afterwards, the electrospun fibrous scaffolds were immersed in IGF1-DA (250 ng/mL), NAT-IGF1(250 ng/mL), Os-DA (50 μg/mL) and NAT-Os (50 μg/mL) solutions for 12 h at 4 °C. Finally, the electrospun fibrous scaffolds were immersed in PBS three times, 5 min for each time, to remove the unbinding proteins and peptides. All the electrospun fibrous scaffolds were freeze-dried for 24 h. Finally, we obtained the electrospun fibrous scaffolds with modified surfaces: (1) PLGA/IGF1-DA, (2) PLGA/NAT-IGF1, (3) PLGA/Os-DA, (4) PLGA/NAT-Os and (5) PLGA/Os-DA/IGF1-DA.

### Characterisation of electrospun fibrous scaffolds with modified surfaces

Scanning electron microscopy (SEM, Zeiss EVO 18, Germany) and multimode scanning-probe atomic force microscopy (AFM, Bruker, Dimension Icon, USA) were used to observe the surface morphology of different groups of electrospun fibrous scaffolds. Fifty fibres were randomly selected to calculate the mean diameter. A drop shape analyser (Kruss, Germany) was used to measure the hydrophilicity of different groups of scaffolds. The tensile strengths of the different groups of fibre scaffolds were measured by a materials-testing machine (Proline, Zwickroell, Germany). The data was calculated and drawn by OriginPro 2017 software.

### Antibacterial assays

*Escherichia coli* (*E. coli*) and *Staphylococcus aureus* (*S. aureus*) were inoculated into a Luria Bertani medium and cultured at 37 °C and 150 rpm to OD_600_ = 0.5. To compare the antibacterial activities against *S. aureus* and *E. coli*, different nanofibrous films were added to tubes with a density of 2.5 × 10^4^ bacteria/mL, and the tubes were incubated overnight at 37 °C and 150 rpm for the bacteria to culture. Then, 100 μl bacteria were spread on agar plates, and the plates were incubated overnight at 37 °C to allow the cells to culture. The number of colonies on different agar plates was calculated using ImageJ software. The bacterial survival ratio was calculated using the following equation:

$$\text{Bacterial survival ratio }=\frac{SG}{\text{CG}} \times 100\%$$

where CG is the number of control group bacterial colonies, and SG is the number of sample group bacterial colonies. The data was calculated and drawn by Origin pro 2017 software.

### Adsorption and release assays

To evaluate the adsorption ability of the PLGA electrospun fibrous scaffolds towards IGF1-DA and NAT-IGF1, different films were immersed into the human IGF1 antibody (1:3000) for 12 h and then immersed in the secondary antibody solution (goat anti-rabbit IgG conjugated with Alexa 488, 1:2000) for 12 h at 4 °C. Then, FITC-Os-DA and FITC-NAT-Os were incubated with the PLGA electrospun fibrous scaffolds and washed three times with PBS. A fluorescence imaging device (CRI Maestro) was used to obtain the fluorescence images of different electrospun fibrous scaffolds. Three images were taken and average signal was measured using software (Maestro 2.4) provided by manufacturers. The data was calculated and drawn by Origin pro 2017 software.

After the different electrospun fibrous scaffolds (1 cm^2^) had absorbed NAT-Os, Os-DA, NAT-IGF1 and IGF1-DA, they were immersed into 5 mL of PBS at room temperature for 3 days, under stirring in a rotary mixer. At 6, 12, 24, 48, 72 h time intervals, peptides and protein content were measured by BCA (Pierce) and ELISA (R&D) kits. The cumulative NAT-Os, Os-DA, NAT-IGF1 and IGF1-DA release rates were calculated and drawn by Origin pro 2017 software.

### Cytocompatibility

BALB/c 3T3 cells were cultured in DMEM (high glucose) containing 10% FBS and penicillin (100 U/mL)-streptomycin (0.1 mg/mL) in a cell incubator (Sanyo, MCO-18AC) at 37 °C with 5% CO_2_. All electrospun fibrous scaffolds were sterilised by soaking them in 75% ethanol for 45 min and then combining them with sterile bioactive factors (0.22 μm filtration). Rubber rings were used to fix the electrospun fibrous scaffolds in a 24-well culture plate. The cells were seeded at a density of 1.2 × 10^5^ on the film surface. On days 3 and 7, the culture medium was changed into a fresh medium containing 10% CCK-8 (*v/v*). After 1 h of incubation, OD_450_ measurements were performed using a microplate reader (Thermo MK3). The data was calculated and drawn by Origin pro 2017 software.To evaluate the morphology and growth density of cells growing on different nanofibrous scaffolds, on day 3, the cells were stained with calcein-AM (2 μM) and incubated at 37 °C for 10 min. The cell morphology was observed and photographed with a fluorescence inverted microscope (Olympus, CKX53).

### Full-thickness wound model and scaffold implantation

Twenty Wistar rats (around 170 g) were used to evaluate wound repair. To assess the ability of the different films to promote wound healing, a round 10 mm-diameter wound was made on the skin of rats. The rats were randomly divided into six groups: (a) control (PLGA), (b) PLGA/NAT-IGF1, (c) PLGA/NAT-Os, (d) PLGA/IGF1-DA, € PLGA/Os-DA and (f) PLGA/Os-DA/IGF1-DA. Four rats were used for each group. The experimental animal protocol was approved by the Animal Experiment Ethics Committee of Jilin University, and all procedures were performed in accordance with the National Regulation of China for Care and Use of Laboratory Animals. After the round wound was made, each group of film was attached to the wound surface. At points 1, 4, 7, 10 and 12, a digital camera (Nikon D750, Japan) was used to photograph the wound. The following equation was used to calculate the wound healing rate of the different groups:

$${\text{Wound healing rate}}= \frac{\left[(-{\text{S}}-{\text{AP}})/{\text{AS}}\right]}{\text{AS}} \times 100\%$$

AS is the area of the postoperative wound, and AP is the area of the wound at different time points after repair. The data was calculated and drawn by Origin pro 2017 software.

### Histology

On day 12, the tissue around the back wound was cut off and immersed in formalin solution for 3 days; afterwards, it was immersed in 75%-, 85%-, 95%-, and 100%-grade alcohol for 30 min for dehydration and then embedded in paraffin and sectioned at 5 μm thickness. All sections were stained with haematoxylin and eosin (H&E) and Masson’s trichrome staining (Masson) and visualised under a light microscope (Zeiss Imager A2, Germany). Epithelial gap and wound closure rate were measured using the ImageJ 1.48v software download from http://imagej.nih.gov/ij. The data was calculated and drawn by Origin pro 2017 software.

### Statistical analysis

Origin 2017 was used for statistical calculations and analysis. One-way analysis of variance and Tukey’s multiple comparison test were used to assess statistical significance. When *p* < 0.05 (*), the data was considered statistically significant. The data were shown as mean ± standard deviation (SD).

## Results and discussion

### Binding and release abilities of IGF1-DA and OS-DA loaded on PLGA electrospun fibrous scaffolds

**Figure 1:**
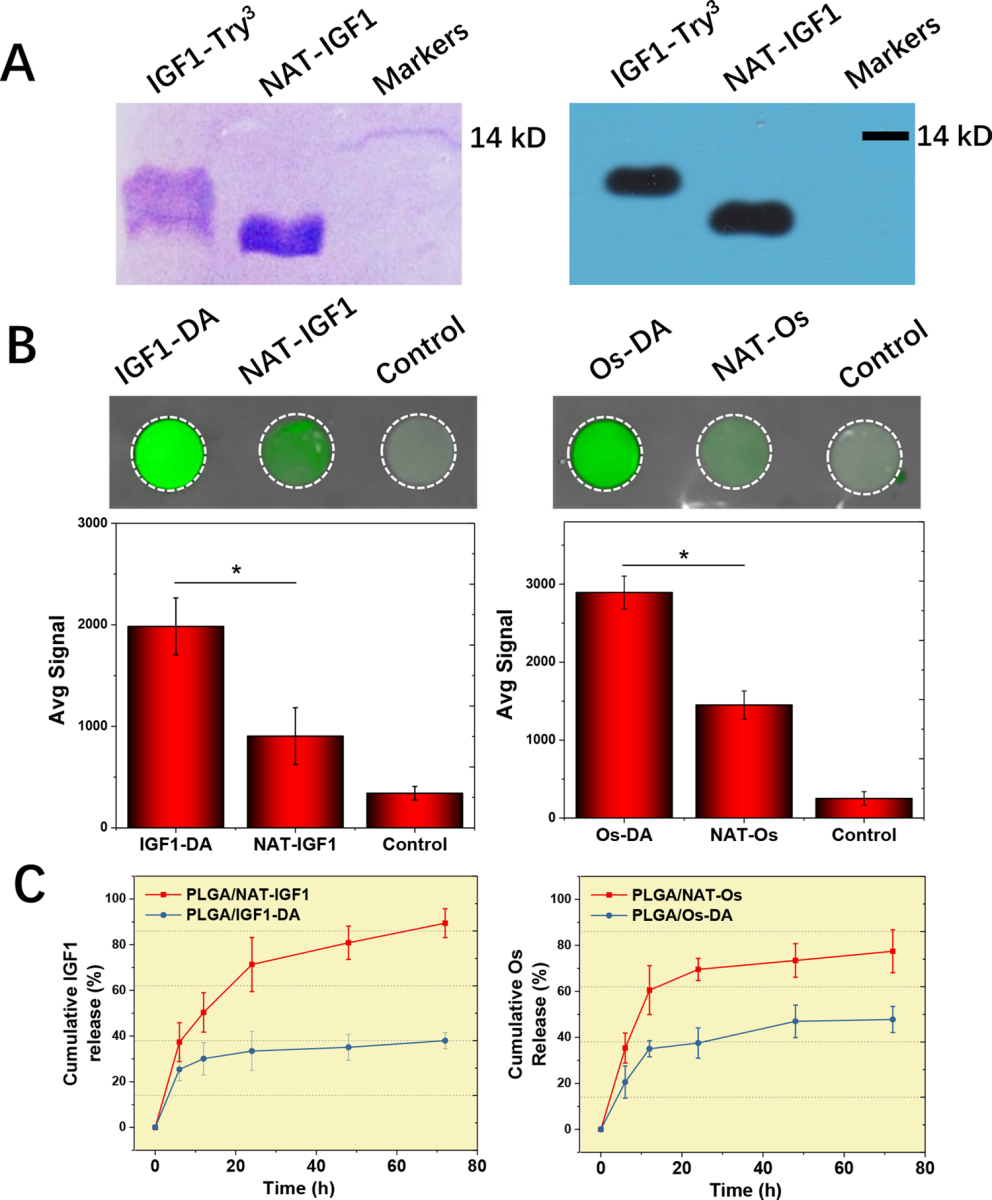
(**A**) Expression and purification of IGF1-Try3 and NAT-IGF1. Recombinant proteins were analyzed using a 15% SDS-PAGE under reducing conditions and western blotting. (**B**) PLGA-binding ability of IGF1-DA, NTA-IGF1, Os-DA and NTA-Os. (**C**) The cumulative release rate during 0 to 72 h, *n* = 3, one-way ANOVA with Tukey’s test was used to determine significance (*p < 0.05).

NAT-IGF1 and IGF1-Try^3^ were successfully obtained by inducing *E. coli* containing NAT-IGF1-PET15b and IGF1-Try^3^-PET15b plasmids, followed by purification with affinity chromatography and desalination chromatography (Fig. 1A). The SDS-PAGE and WB results showed that the molecular weights of NAT-IGF1 and IGF1-Try^3^ were basically consistent with the theoretical molecular weights (NAT-IGF1 = 9.87 kD, IGF1-Try^3^ = 10.61 kD). The protein purity was above 95%. Intact SDS-PAGE electrophoresis and WB images of IGF1-Try^3^ and NAT-IGF1 were shown in Fig. S1 and Fig. S2, respectively.

Wound regeneration requires the participation and coordination of various growth factors. At the same time, there is a risk of bacterial infection, as the skin that protects against bacterial infections from the external environment is damaged. Inspired by the DA molecule in mussel adhesion protein, many studies have been conducted to obtain the sustained release ability of growth factors at the wound site. In the current study, the repeat sequence Tyr–Lys–Tyr–Lys–Tyr containing tyrosine was added to the end of the growth factor via recombinant gene technology, and tyrosine was transformed into a DA molecule through in vitro hydroxylation. To enhance the adhesion ability of the antibacterial polypeptide OS, we synthesised the OS polypeptide containing the Tyr–Lys–Tyr–Lys–Tyr tag. The same method could also endow the OS with strong adhesion ability. The fluorescence imaging results (Fig. 1B) showed that Os-DA and IGF1-DA had a stronger binding ability to PLGA films than NAT-Os peptide and NAT-IGF1. This result indicates that we successfully obtained Os and IGF1 containing DA molecules, and the DA molecules in the Os-DA and IGF1-DA showed a strong binding ability to the material surface.

Polymer materials lack active factor binding sites, which makes it difficult for bioactive factors to bind to the materials. Therefore, the release of the bioactive factors loaded on the material surface can easily occur, resulting in unnecessary waste and side effects. Therefore, improving the binding ability of bioactive factors to the dressing materials and ensuring their stable release is of great importance. As shown in Fig. 1C, after a 72 h release experiment, we tested the sustained release of Os-DA and IGF1-DA. During the whole 72 h release process, both Os and IGF1 containing DA molecules exhibited slow release, which was mainly related to the DA molecule ability to adhere to the material surface. The cumulative release rates of IGF1-DA and Os-DA during the 72 h period were 37.98% and 47.75%, respectively, while those of the NAT-IGF1 and NAT-Os were 89.41% and 77.42%, respectively. These results fully demonstrate the effect of DA introduction on the sustained release ability of growth factors and polypeptides. The sustained release of growth factors can promote cell proliferation and differentiation and consequently accelerate wound healing. The sustained release of antibacterial polypeptides (NAT-Os) can effectively inhibit bacterial infection, thereby reducing the inflammatory response at the wound site and accelerating wound healing.

### In vitro antibacterial tests

**Figure 2:**
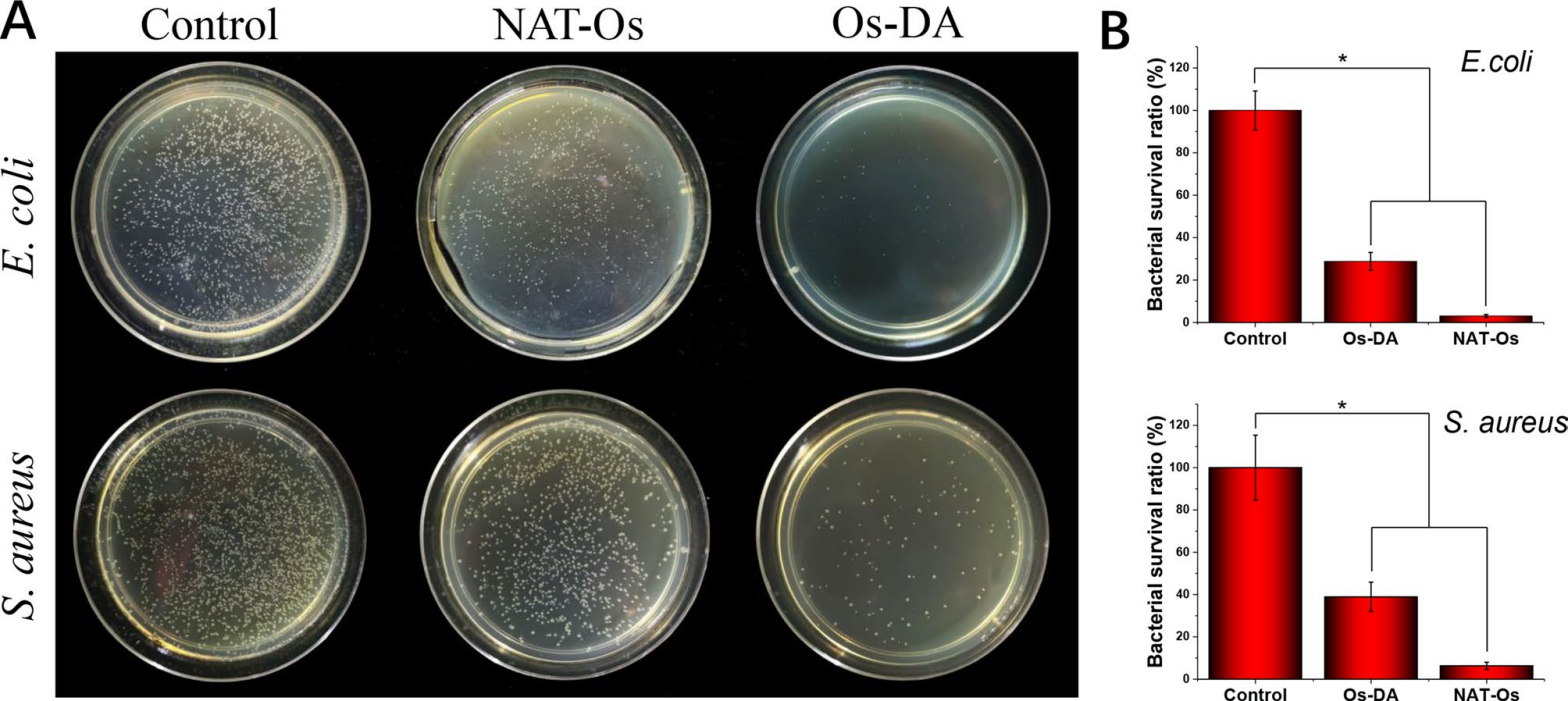
Antibacterial activity of PLGA electrospun fibrous scaffolds coated with NAT-OsandOs-DA. (**A**) Photographs of bacterial colonies formed by *E. coli* and *S. aureus* cells treated withPLGA/NAT-Os and PLGA/Os-DA. (**B**) Bacterial survival ratio of different group, *n* = 3, one-way ANOVA with Tukey’s test was used to determine significance (*p < 0.05).

To effectively accelerate the skin wound healing process, the dressing material should have excellent antibacterial properties and inhibit bacteria growth. However, pure PLGA polymer cannot effectively inhibit bacteria growth, which severely limits its application in the field of wound repair. Therefore, in this study, we used DA-Os to modify the surface of PLGA electrospun fibrous scaffolds to improve its antibacterial properties. To test the antibacterial performance of different electrospun fibrous scaffolds, *E. coli* and *S. aureus* were co-cultured with PLGA, PLGA/NAT-Os and PLGA/DA-Os electrospun fibrous scaffolds, and the bacterial survival rates were measured and observed. Since PLGA had no antibacterial activity, it was the control group. As shown in Fig. 2, significant bacterial growth, which almost covered the entire plate, was observed in the PLGA group. In comparison, much fewer bacterial colonies were found on the PLGA/NAT-Os, due to the addition of Os. The results show that the AMPs surface modification can significantly improve the polymer materials antibacterial activity. Moreover, after the PLGA was loaded with DA-Os, the bacterial colonies of the PLGA/DA-Os group were further reduced compared with that of the pure PLGA/NAT-Os group, and colonies could hardly be observed on the plate surface for the former group. This may be due to the excellent adhesion ability of DA, which could bind the AMPs more tightly to the PLGA material, preventing the removal and activity destruction of AMPs. Finally, we calculated the bacterial survival rate of each sample. The bacterial survival rates of *E. coli* and *S. aureus* in the PLGA/NAT-Os group were 28.81% and 38.88%, respectively. Those for the PLGA/DA-Os group were lower: 3.05% and 6.30%, respectively. The above bacteriostatic experiment results indicate that the introduction of DA molecules into the antibacterial peptide terminal of Os can significantly enhance its load on PLGA electrospun fibrous scaffolds and maximise the antibacterial properties of the dressing material.

### Characterisation of electrospun fibrous scaffolds

**Figure 3:**
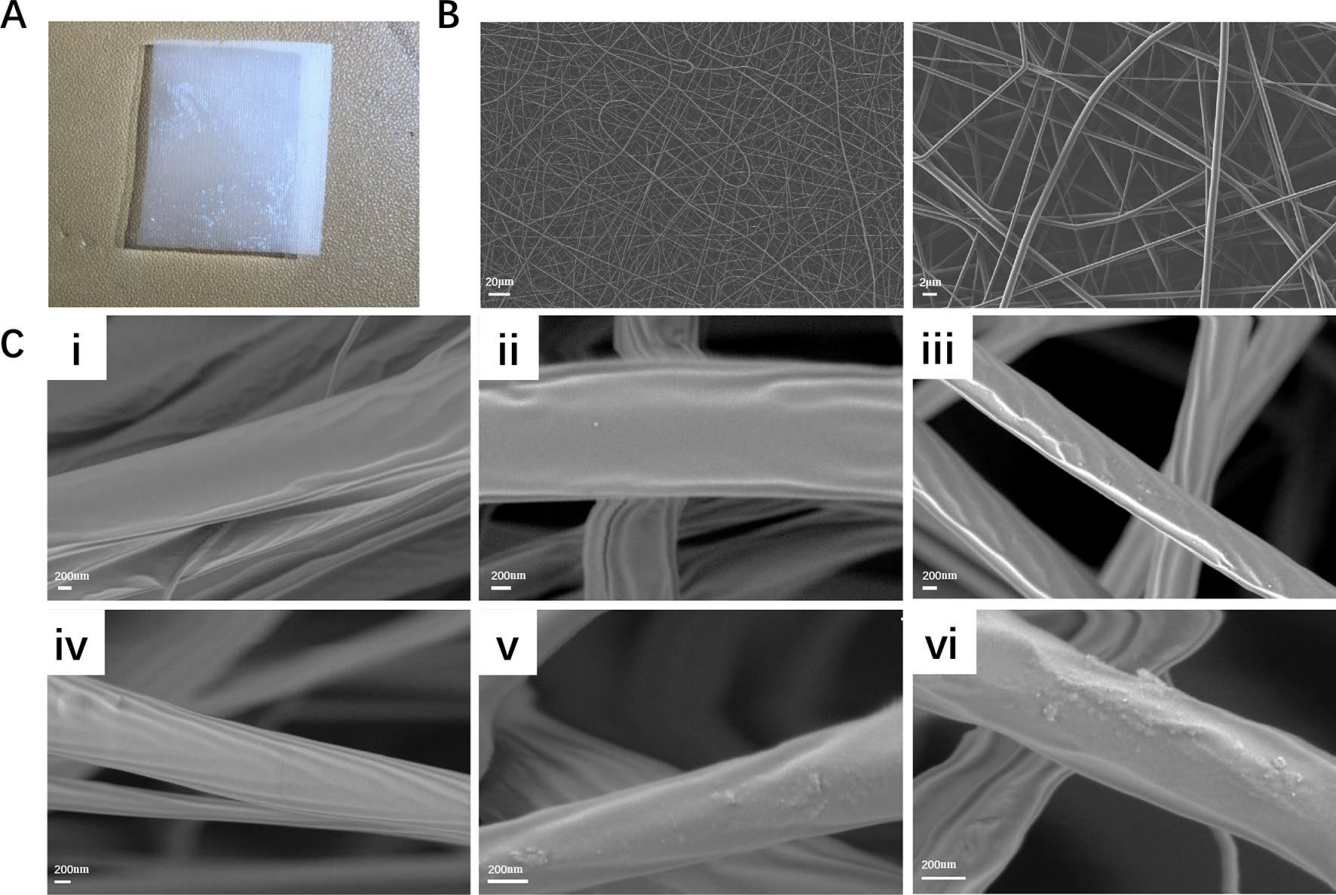
(**A**) Photograph of the electrospun nanofiber film. (**B**) SEM micrographs of the electrospun nanofiber film. (**C**) Surface morphology of (i) control, (ii) PLGA/NAT-Os, (iii) PLGA/Os-DA, (iv) PLGA/NAT-IGF1, (v) PLGA/IGF1-DA and (vi) PLGA/Os-DA/IGF1-DA electrospun fibrous scaffolds.

**Figure 4:**
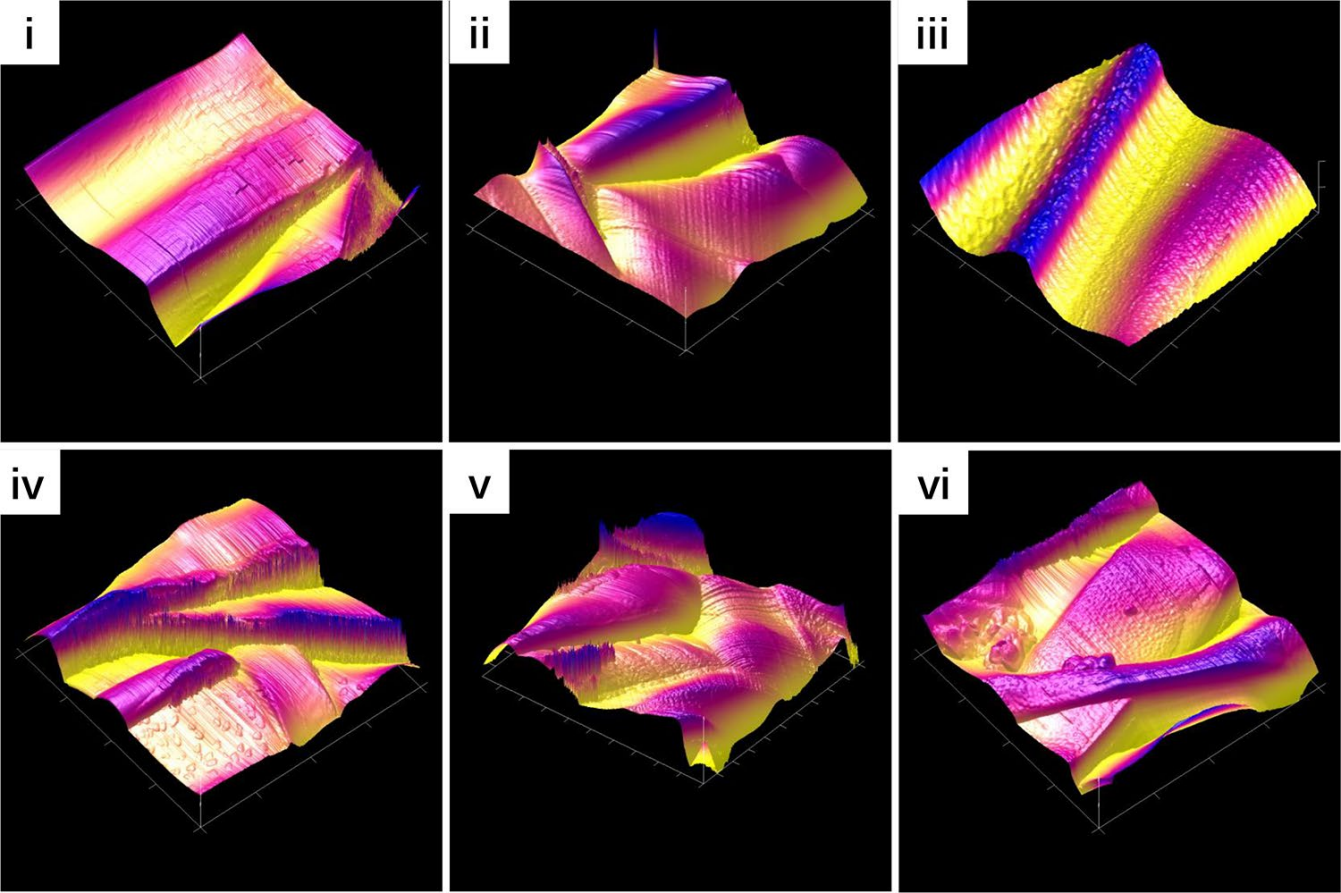
AFM images of (i) control, (ii) PLGA/NAT-Os, (iii) PLGA/Os-DA, (iv) PLGA/NAT-IGF1, (v) PLGA/IGF1-DA and (vi) PLGA/Os-DA/IGF1-DA electrospun fibrous scaffolds; scale size for AFM: 2 × 2 μm.

The surface microstructure is a vital parameter for the electrospun fibrous scaffold characterisation, as it has important effects on cellular behaviour such as cell adhesion and proliferation. As seen in Fig. 3A,B, a 3D network structure with the topological structure of natural ECM was fabricated by electrospinning; such a structure can create a good cellular microenvironment for cell adhesion and growth. The diameter of the different electrospun fibrous scaffolds ranged from 550 to 1100 nm. Figure 3C shows the SEM images of untreated PLGA, PLGA/NAT-IGF1, PLGA/NAT-Os, PLGA/IGF1-DA, PLGA/Os-DA and PLGA/Os-DA/IGF1-DA electrospun fibrous scaffolds. The untreated PLGA nanofiber had a uniform structure with a smooth surface. However, the other nanofibers featured a rough surface, which was possibly due to the IGF1 or Os layer immobilisation. Furthermore, the DA-modified bioactive factor-grafted electrospun fibrous scaffold had a rougher surface than the unmodified bioactive factor-grafted electrospun fibrous scaffold. The rougher surface morphology could be due to the immobilisation of more DA-modified bioactive factors on the nanofiber surface. More importantly, the growth factor and AMPs surface modification process did not deform the nanofiber structure of the PLGA electrospun fibrous scaffold, as evident in the SEM images. AFM was further used to study the more detailed structure and surface morphology of the electrospun fibrous scaffolds. Figure 4 shows typical 3D AFM images of each of the mats over an area of 2 × 2 μm. The smoothest surface belonged to the PLGA electrospun fibrous scaffold, whereas the roughest belonged to the DA-modified bioactive grafted electrospun fibrous scaffold. Previous studies have found that rough surfaces are more conducive to cell adhesion and migration^27^. Thus, the surface structure of the DA-modified bioactive grafted electrospun fibrous scaffold would provide a better environment for cell growth.

### Hydrophilicity and mechanical properties assessment

**Figure 5:**
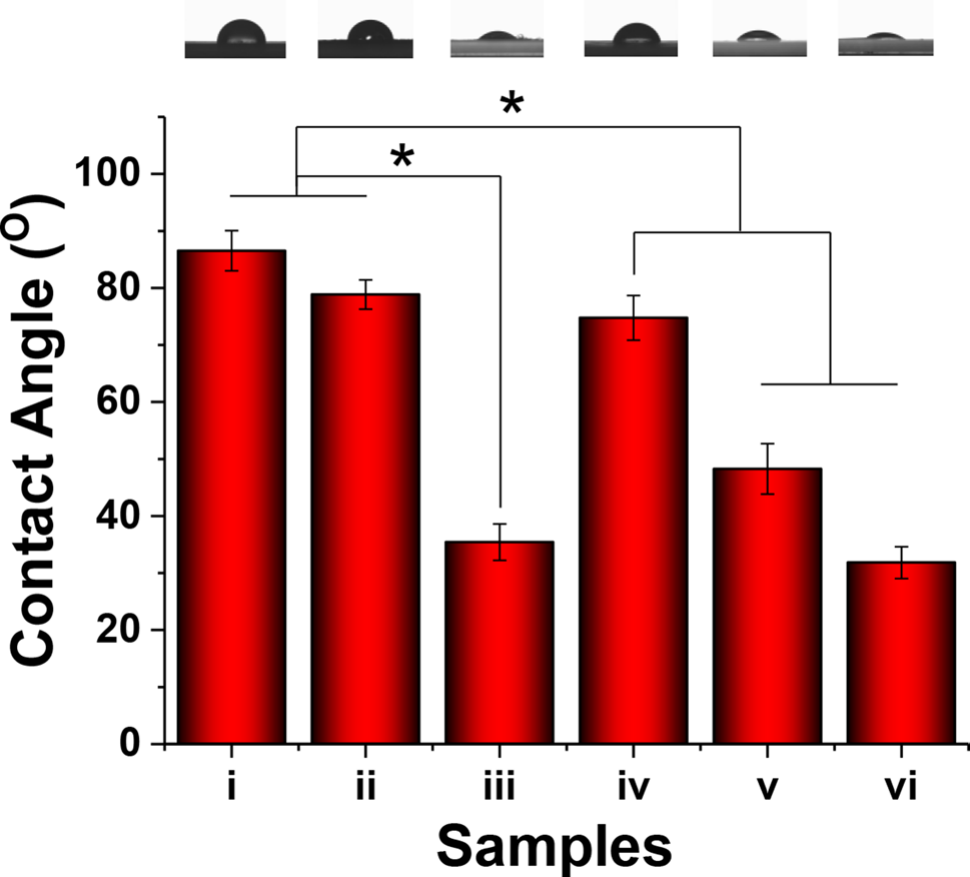
Wettability assessment of (i) control, (ii) PLGA/NAT-Os, (iii) PLGA/Os-DA, (iv) PLGA/NAT-IGF1, (v) PLGA/IGF1-DA and (vi) PLGA/Os-DA/IGF1-DA electrospun fibrous scaffolds, *n* = 3, one-way ANOVA with Tukey’s test was used to determine significance (* *p* < 0.05).

**Figure 6:**
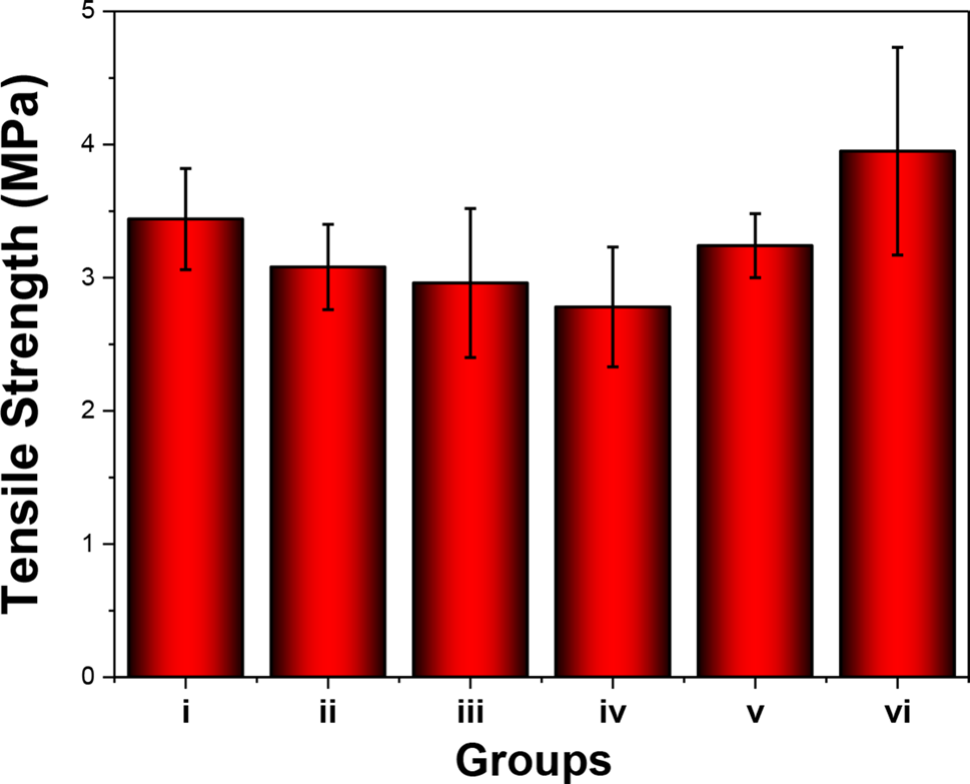
Tensile strengths of different nanofibrous films at σc 20%. (i) Control, (ii) PLGA/NAT-Os, (iii) PLGA/Os-DA, (iv) PLGA/NAT-IGF1, (v) PLGA/IGF1-DA, (vi) PLGA/Os-DA/IGF1-DA electrospun fibrous scaffolds, *n* = 3, one-way ANOVA with Tukey’s test was used to determine significance (*p < 0.05).

Previous studies have shown that the hydrophilicity of biomaterials is crucial for cell growth^30,31^. In the current study, the water contact angle on the electrospun fibrous scaffold was measured to obtain the relative hydrophobicity and hydrophilicity of the surfaces before and after the growth factor and AMP surface modification. The PLGA sample exhibited a higher contact angle (96.53 ± 3.52°) than the other groups (Fig. 5). This high contact angle indicates that the PLGA surface was hydrophobic. After IGF1 and Os were immobilised on the surface, the contact angles of the electrospun fibrous scaffolds slightly decreased. Among all electrospun fibrous scaffolds, the DA-modified bioactive factors-grafted electrospun fibrous scaffold featured the best hydrophilicity. The water contact angles for the different scaffolds were as follows: 48.27 ± 4.43° (PLGA/Os-DA), 35.43 ± 3.19° (PLGA/IGF1-DA), and 31.83 ± 2.78° (PLGA/Os-DA/IGF1-DA). The PLGA electrospun fibrous scaffold achieved superhydrophilicity by IGF1-DA and Os-DA surface modification. This phenomenon demonstrates that DA, which contains hydrophilic groups, was successfully introduced into the bioactive factor. Many studies have found that DA can make the surface of polymer materials more hydrophilic, due to the presence of various hydrophilic groups in its structure, such as catechol, quinone and amine^32,33^. We also tested the tensile strengths of the different electrospun fibrous scaffolds. The tensile strength at σc 20% of the PLGA sample was approximately 3.44 ± 0.38 MPa (Fig. 6). There was no significant difference in tensile strength between the PLGA sample and the other bioactive factor-modified electrospun fibrous scaffolds, which indicates that the bioactive factor surface modification process does not destroy the inherent properties of the electrospun fibrous scaffold. The above results show that the adopted DA-modified bioactive factor surface modification is simple, stable, effective and mild; thus, it can overcome the shortcomings of surface hydrophobicity of polymer materials and has no harmful effect on mechanical and structural properties.

### Cell adhesion and proliferation

**Figure 7:**
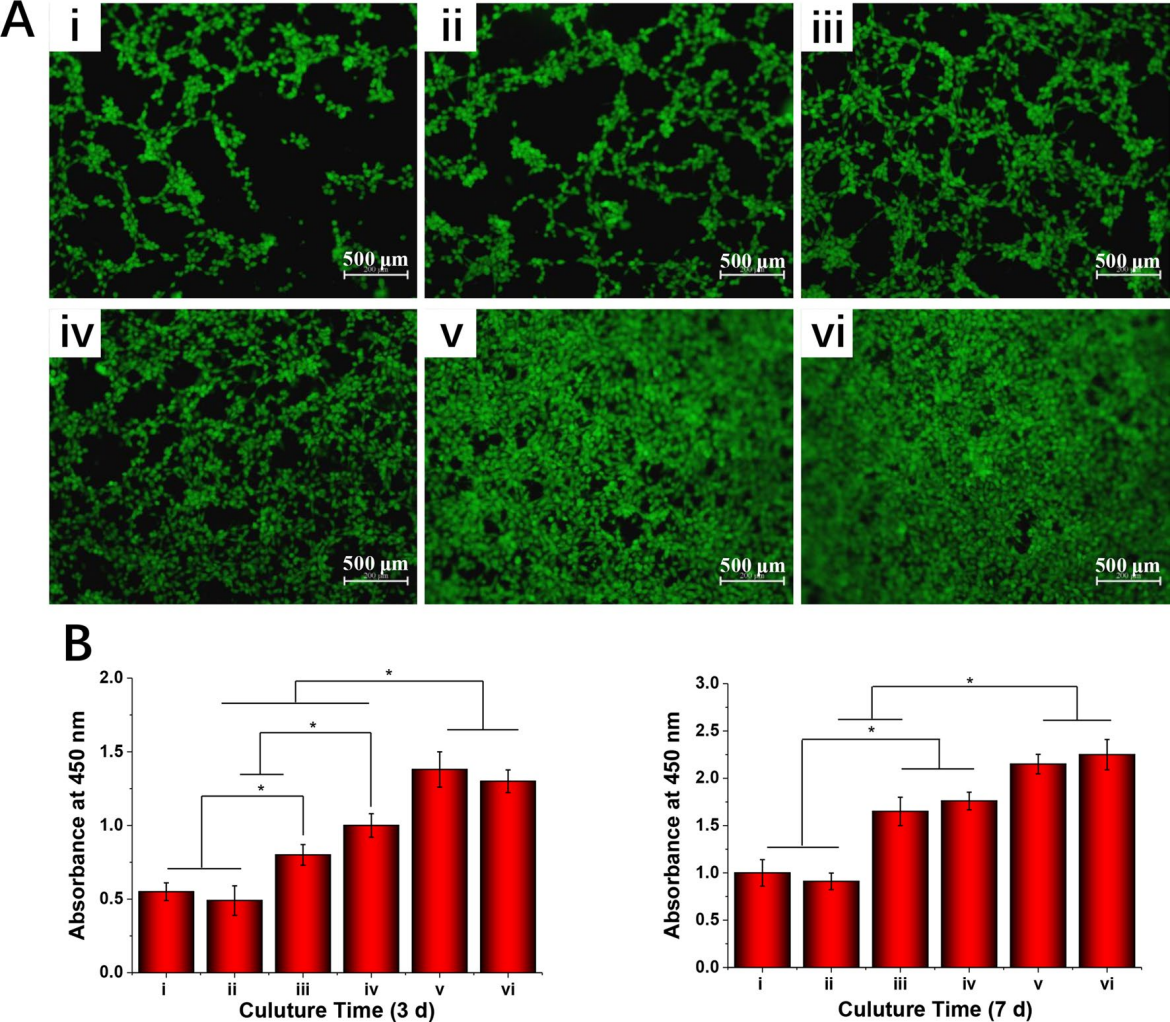
(**A**) Cultured cell morphology at 3 days stained by Calcein AM. (**B**) Proliferation of cultured cell at 3 and 7 daysdetected using CCK-8 kit, (i) PLGA, (ii) PLGA/NAT-Os, (iii) PLGA/Os-DA, (iv) PLGA/NAT-IGF1, (v) PLGA/IGF1-DA, (vi) PLGA/Os-DA/IGF1-DA, *n* = 4, one-way ANOVA with Tukey’s test was used to determine significance (*p < 0.05).

To better observe the effect of the different electrospun fibrous scaffolds on cell adhesion, BALB/C 3T3 cells were cultured in the electrospun fibrous scaffolds. After 3 days of culture, the cells morphology was observed with a fluorescence microscope. The BALB/C 3T3 cells presented good attachment, spreading and growth after 3 days of incubation (Fig. 7A), suggesting that skin fibroblasts could adhere to the surfaces of the different scaffolds. Furthermore, the cells on the PLGA/NAT-IGF1 scaffold exhibited a greater spread, with a better cytoskeleton, than those on the PLGA and PLGA/NAT-Os scaffolds. Among all samples, the IGF1-DA-modified electrospun scaffolds showed the most uniform distribution of the adhered cells; some cells bridged across each other formed a confluent cell monolayer. These results indicate that the IGF1-DA can change the PLGA surface performance and promote cell proliferation and adhesion.

The basic property requirement of biomaterials is good compatibility. Therefore, the biocompatibilities of the electrospun fibrous scaffolds were evaluated based on cell proliferation and cell adhesion. The total BALB/C 3T3 cell populations grown on the different electrospun fibrous scaffolds after 3 and 7 days were detected using CCK-8 assay, and the results are shown in Fig. 7B. No significant differences in cell proliferation existed between the PLGA and PLGA/NAT-Os scaffolds, for both growth periods of 3 and 7 days, demonstrating the nontoxicity of the NAT-Os. Previous studies have shown that NAT-Os has good cellular compatibility and will not have any negative effect on eukaryotic cells^24^. The results of these studies are similar to the cell proliferation result in this study. Furthermore, the PLGA/NAT-IGF1 scaffold was found capable of promoting cell proliferation more effectively than the PLGA and PLGA/NAT-Os scaffolds. Previous studies have demonstrated that NAT-IGF1 can significantly promote cell proliferation as a therapeutic factor either alone or in combination with other active ingredients^20,22,27^. More importantly, compared with the PLGA/NAT-IGF1 and PLGA/NAT-Os scaffolds, the PLGA/IGF1-DA and PLGA/Os-DA scaffolds presented a higher OD value, and the cell proliferation increased with the addition of the DA-modified growth factor. Thus, the DA-modified bioactive factor can effectively enhance the scaffold biocompatibility. We speculate that the rough surface and excellent hydrophilicity can improve the cell affinity of the polymer materials. Furthermore, DA contains a large number of amines and hydroxyl groups, which also creates a good environment for cell growth.

### Wound healing

**Figure 8:**
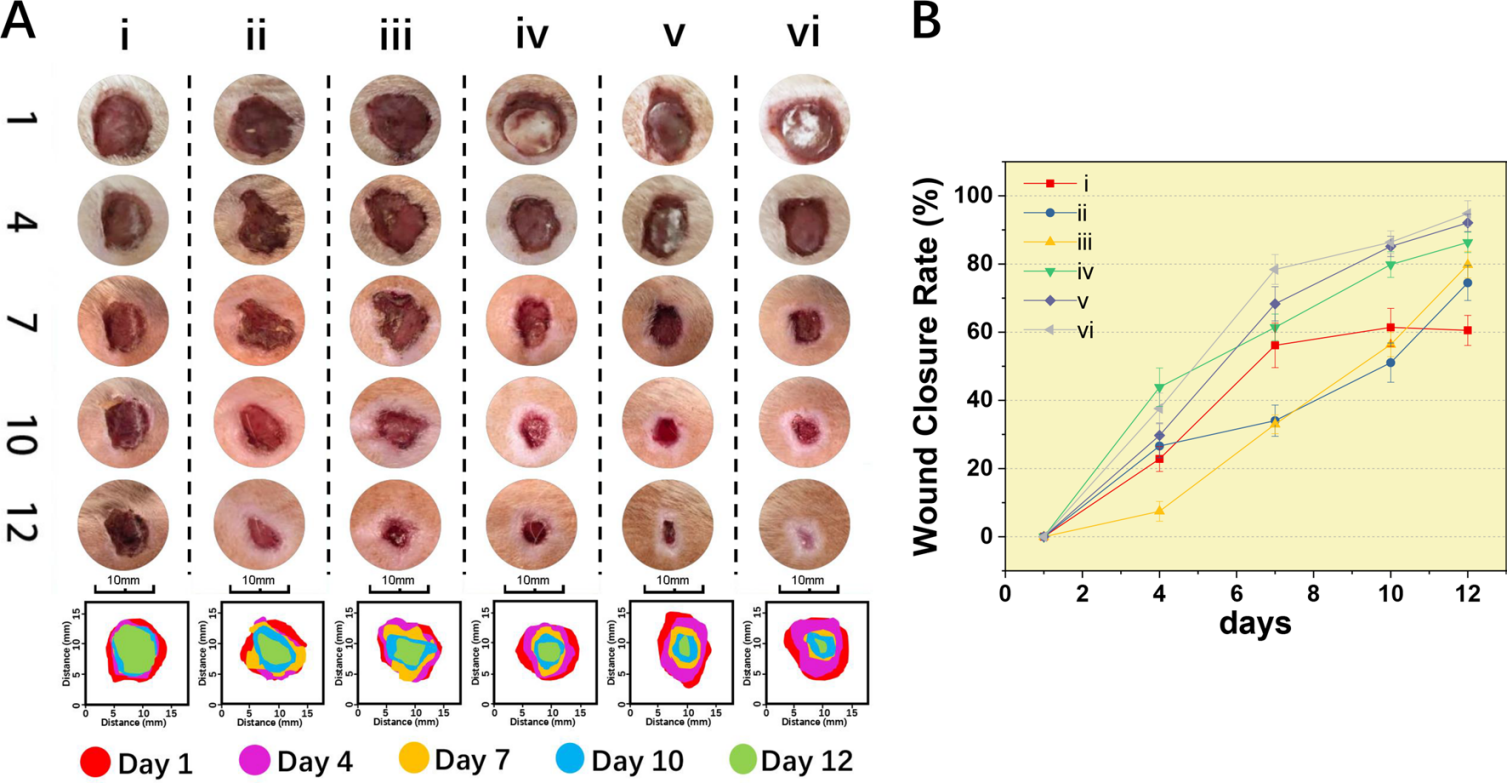
(**A**) The comparative assessment of the gross appearance of wound healing on different treated groups for different experimental days 1, 4, 7, 10 and 12 in rats. (**B**) wound closure rate from 1 to 12 days; (i) control, (ii) PLGA/NAT-Os, (iii) PLGA/Os-DA, (iv) PLGA/NAT-IGF1, (v) PLGA/IGF1-DA, (vi) PLGA/Os-DA/IGF1-DA electrospun fibrous scaffolds, *n* = 4.

**Figure 9:**
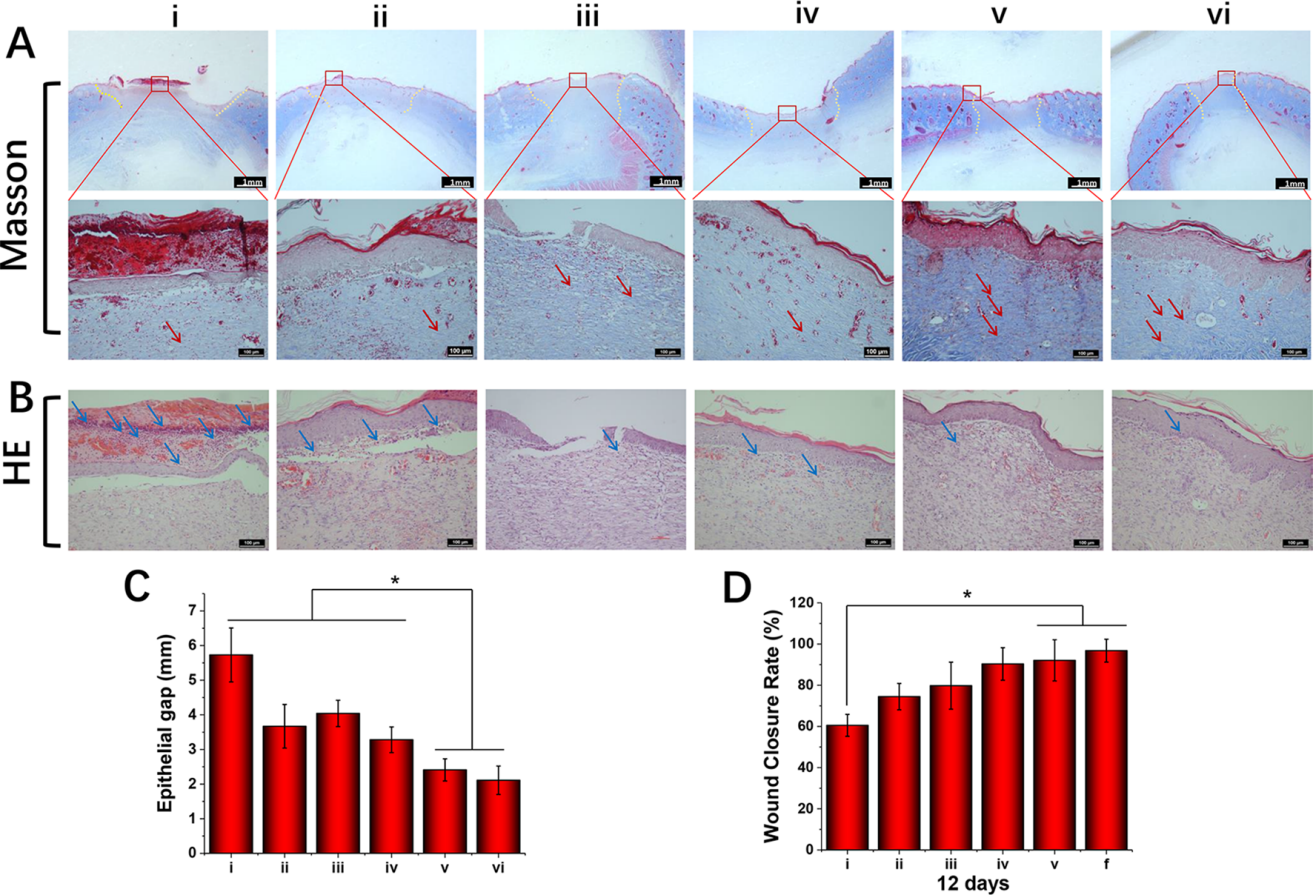
Histological staining evaluation of wound regeneration for (i) PLGA, (ii) PLGA/NAT-Os, (iii) PLGA/Os-DA, (iv) PLGA/NAT-IGF1, (v) PLGA/IGF1-DA and (vi) PLGA/Os-DA/IGF1-DA electrospun fibrous scaffolds: (**A**) masson staining images, yellow dotted lines indicate the epithelial gaps, and red arrows indicate collagen fibres; (**B**) H&E staining images, blue arrows indicate neutrophils; (**C**) epithelial gaps; (**D**) wound closure rate on day 12, *n* = 4, one-way ANOVA with Tukey’s test was used to determine significance (*p < 0.05).

To evaluate the potential clinical applications of the different electrospun fibrous scaffolds, the tissue repair ability of the electrospun fibrous scaffold was further examined by an in vivo test. As shown in Fig. 8, the wound area was measured daily for 12 days. On day 7, the wound area of all groups decreased to some extent. Among the scaffolds, PLGA/NAT-IGF1 (61.40% ± 2.75), PLGA/IGF1-DA (68.32% ± 7.92) and PLGA/Os-DA/IGF1-DA (78.40% ± 9.01) exhibited the minimum wound areas, which demonstrated its comparatively higher promotion effect on wound healing. The growth factor IGF1 plays a crucial role in different stages of wound healing (inflammation, proliferation and remodelling); for example, it helps in removing reactive oxygen species and increasing the antioxidant enzyme production around wounds^31^. Specifically, on day 12, wounds treated with PLGA/IGF1-DA and PLGA/Os-DA/IGF1-DA scaffolds showed the smallest areas (90.35% and 92.08%) (Fig. 9D). Furthermore, the wound area analysis showed that the wound healing effects of PLGA/NAT-Os and PLGA/Os-DA groups were better than that of the PLGA group, due to the inherent antibacterial properties of Os. When the electrospun fibrous scaffolds were modified with both IGF1-DA and Os-DA, the scaffolds showed a more effective therapeutic effect in the whole wound healing process. These results demonstrate that PLGA/Os-DA/IGF1-DA electrospun fibrous scaffolds have good biological properties and can effectively improve the wound healing rate in full-thickness skin defect models.

### Histological analysis

We used Masson’s trichrome staining to evaluate the results of collagen deposition. Blue collagen fibre deposition was observed in each group, with that in the PLGA group being the least significant (Fig. 9A). After the growth factor was immobilised, the collagen fibre production was significantly improved; this phenomenon was more remarkable in the PLGA/IGF1-DA and PLGA/Os-DA/IGF1-DA groups than the PLGA/NAT-IGF1, suggesting that the DA-modified growth factor can more effectively promote collagen formation. The obtained experimental results demonstrate that the PLGA/Os-DA/IGF1-DA electrospun fibrous scaffolds showed good biocompatibility in vitro; moreover, it can effectively promote skin wound healing and collagen deposition.

The histological examination of the groups treated with different electrospun fibrous scaffolds was carried out using H&E on day 12 of the post-operational period, and the results are illustrated in Fig. 9B. Most of the wound surfaces treated with PLGA/NAT-IGF1, PLGA/IGF1-DA and PLGA/Os-DA/IGF1-DA groups were replaced by new normal skin tissues, and the keratinocytes layer on the wound external surface was relatively dense. In contrast, the skin layers treated with PLGA group were thick, incompact and incomplete. Unlike in the wound treated by the PLGA group, a uniform thickness of the new epidermis, hair follicles and well-proliferated fibroblasts were formed at the edge of the wound treated by the PLGA/Os-DA group; this demonstrates that the antibacterial polypeptide can contribute to maintaining bio-balance and wound healing. We also measured the defective width and epithelial gap of skin sample sections. The longest defect width was found in the PLGA group, while the shortest defect width was found in the PLGA/IGF1-DA and PLGA/Os-DA/IGF1-DA groups (Fig. 9A), indicating that the wound epithelialisation rate was effectively improved by the electrospun fibrous scaffolds. The same results were found in the epithelial gap analysis (Fig. 9C); the PLGA/IGF1-DA and PLGA/Os-DA/IGF1-DA treatments significantly reduced the size of the epithelial gap, indicating accelerated wound closure compared with the cases corresponding to the other groups. Based on the above results, the PLGA/Os-DA/IGF1-DA electrospun fibrous scaffold has a better wound repair ability than the other scaffolds and can effectively promote skin tissue regeneration and collagen deposition.

To prepare ideal skin dressing materials, many factors should be considered, such as biocompatibility, mechanical properties, antimicrobial activity and repair effect. Among the problems in wound therapy, bacterial infections and poor wound healing effect are the foremost. Many previous studies have used biodegradable polymer materials combined with growth factors or drugs to repair skin wounds^34–36^. The repair effect and antibacterial activity of skin dressings can be effectively enhanced by antibacterial agents and growth factors. However, the traditional ways of combining bioactive factors with polymer materials involve many problems, such as a weak binding ability, complex operation and low retention of growth factors at the relevant site. To overcome these issues, we introduced the site-specific DA into IGF1 and Os using recombinant DNA technology, and IGF1-DA and Os-DA were applied for the surface modification of PLGA electrospun fibrous scaffolds. Our data show that compared with unmodified bioactive factors, IGF1-DA and Os-DA can more efficiently bind to PLGA electrospun fibrous scaffolds. The in vivo results show that the PLGA/Os-DA/IGF1-DA electrospun fibrous scaffold can promote skin wound healing and significantly reduce infection rates in surrounding tissues. Therefore, surface modification with recombinant bioactive factors is a promising approach to improve the repair efficiency and antibacterial activity of skin dressing materials. In sum, PLGA/Os-DA/IGF1-DA electrospun fibrous scaffolds are promising candidate materials for skin tissue engineering.

## Conclusions

In this study, we used novel binding bioactive factors (IGF1-DA and Os-DA) to improve the tissue repair effect and antimicrobial activity of PLGA electrospun fibrous scaffolds. The bioactive factors IGF1-DA and Os-DA could more effectively bind to PLGA scaffold and thus improve its therapeutic efficiency. Moreover, the DA-modified bioactive factors could effectively improve the hydrophilicity and biocompatibility of the electrospun fibrous scaffolds surface, which led to increased cell proliferation and adhesion. Furthermore, antibacterial experiments confirmed that the activity of Os-DA was well maintained in the electrospun fibrous scaffolds and could effectively inhibit the growth of *E. coli* and *S. aureus*. In vivo full-thickness wound healing experiments in rats showed that PLGA electrospun fibrous scaffolds with IGF1-DA and Os-DA can promote skin wound healing and collagen deposition, which can potentially provide better therapeutic effect for skin wound treatment. The results show that the presented PLGA/Os-DA/IGF1-DA electrospun fibrous scaffold has application potential for skin wound treatment.

## Supplementary Information

Supplementary Figures.
