# Supplementary material for: Dual bio-active factors with adhesion function modified electrospun fibrous scaffold for skin wound and infections therapeutics
Source: Sci Rep. 2021 Jan 11;11:457. doi: 10.1038/s41598-020-80269-2 (PMC7801708; doi:10.1038/s41598-020-80269-2)
Supplement: Supplementary file 1 — Supplementary Figures. [file 41598_2020_80269_MOESM1_ESM.docx]

# Supplementary Information

# [Dual bio](https://vpns.jlu.edu.cn/http/77726476706e69737468656265737421f1e7518f69276d52710e82a297422f30a0c6fa320a29ae/full_record.do?product=UA&search_mode=GeneralSearch&qid=2&SID=5CD13VyZkGOYhZOkfjX&page=1&doc=1&cacheurlFromRightClick=no)-active factors with adhesion function modified electrospun fibrous scaffold for skin wound and infections therapeutics

Jianhang Jiao^1^, Chuangang Peng^1^, Chen Li^1^, Zhiping Qi^1^, Jing Zhan^2^, Su Pan^1, *^

1. Department of Orthopedic Surgery, The Second Hospital of Jilin University, Ziqiang Street No. 218, Changchun, 130041, Jilin, PR China
2. Department of Gastroenterology, First Hospital of Jilin University, Jilin University, 71 Xinmin Street, Changchun, 130021, Jilin, PR China

*Corresponding author: Su Pan

TEL/Fax: 86- 13504311927

Email: [pansu@jlu.edu.cn](mailto:pansu@jlu.edu.cn)


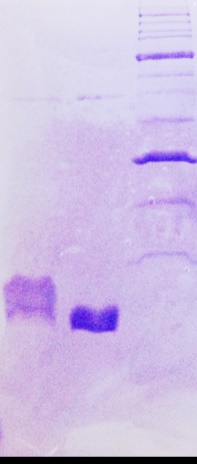


a b M

Fig.S1 IGF1- Try3 (a) and NAT-IGF1(b) SDS-PAGE electrophoretogram.


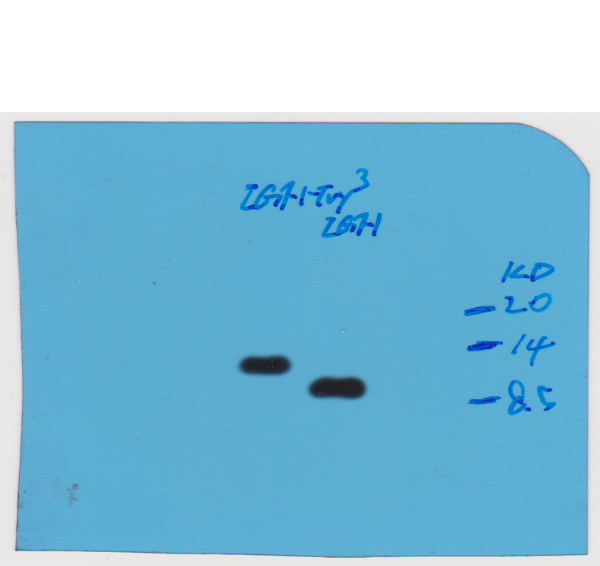


a b

Fig.S2 IGF1- Try3 (a) and NAT-IGF1(b) western blot.
